# Varied Presentations and Comorbidities in Pediatric Autoimmune Pancreatitis

**DOI:** 10.1097/PG9.0000000000000035

**Published:** 2020-12-09

**Authors:** Blair Suter, Feenalie Patel, Kathleen Holland, Brandon P. Brown, Heli Bhatt, Kanika Puri, Brian McFerron, Charles Vanderpool

**Affiliations:** From the Department of Internal Medicine and Pediatrics, Riley Hospital for Children, Indianapolis, IN.

**Keywords:** pediatrics, autoimmune pancreatitis, INternational Study Group of Pediatric Pancreatitis, In search for a cuRE (INSPPIRE)

## Abstract

Autoimmune pancreatitis (AIP) is a chronic inflammatory condition rarely reported in children. In 2018, to standardize the approach to AIP, INternational Study Group of Pediatric Pancreatitis: In search for a cuRE (INSPPIRE) defined AIP, outlined the clinical course, and developed diagnostic and therapeutic recommendations. We performed a retrospective review of cases at our institution from January 1, 2016, to June 1, 2019, and compared their presentations with the INSPPIRE guidelines. Our patients showed variable laboratory, radiographic, and histologic findings, highlighting the difficulty in diagnosing AIP. Histologic samples were obtained in our patients due to diagnostic uncertainty, which ultimately confirmed the diagnosis. One patient was diagnosed with autoimmune hepatitis coexistent with AIP, which has not been previously described in the pediatric population. Exocrine and endocrine complications of AIP were also noted. In all cases, symptoms improved following treatment, and decompression of the common bile duct was seen on repeat imaging.

Autoimmune pancreatitis (AIP) is a chronic inflammatory condition rarely reported in children. Cases of AIP at a single academic center were reviewed from January 01, 2016, to June 01, 2019. The aim of this report is to describe differences in presentation and diagnostic considerations of pediatric AIP while illustrating comorbid conditions that can occur.

## CASE REPORT

### Patient 1

**FIGURE 1.**
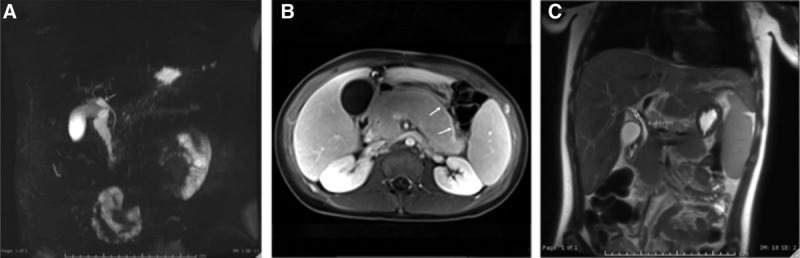
Magnetic resonance cholangiopancreatography (MRCP) of patients 1, 2, and 3. A, Patient no. 1 is a coronal 3D MRCP image. B, Patient no. 2 is an axial T1 post-contrast delayed (120 seconds) image, and (C) patient no. 3 is a coronal single-shot fast spin-echo image. Provided MR images display effects of chronic autoimmune pancreatitis, including dilatation of the CBD, delayed pancreatic capsular enhancement, and patchy parenchymal enhancement. Additional characteristic features of AIP with full descriptions found in Table 1.

A 13-year-old male presented with 4 days of abdominal pain. Investigations showed a normal comprehensive metabolic panel and lipase elevation of 92 Units/L (7–59 Units/L). Transabdominal ultrasound (TUS) revealed common bile duct (CBD) dilatation of 9 mm. Magnetic resonance cholangiopancreatography (MRCP) was concerning for AIP versus atypical acute interstitial pancreatitis, with findings of enlarged pancreatic head/uncinate process and tail with patchy areas of delayed enhancement and focal dilatation of the extrahepatic CBD (Fig. 1A). Additional testing showed elevated IgG of 1620 mg/dL (680–1531 mg/dL) and IgG4 of 163 mg/dL (4–136 mg/dL).

**Table 1.** Imaging and Histology Between Patients and INNSPIRE Definitions

**Patient**	**TUS**	**MRCP**	**ERCP/EUS**	**Histology**
**1**	CBD dilatation of 9 mm	Edematous enlargement of pancreatic head/uncinate process and tail, with associated patchy areas of delayed enhancement and abnormal focal dilatation of the extrahepatic CBD.	Hyperechoic strands, hypoechoic foci, and lobularity throughout the pancreatic parenchyma and dilatation of the common hepatic duct of 8 mm due to edema of the pancreatic head	Needle core biopsies of the pancreas showed acute inflammation and patchy mild chronic inflammation with neutrophils within ductal structures and acinar epithelial cells consistent with granulocytic epithelial lesions. There was no increase in cells staining with IgG4; there were scattered plasma cells positive for CD138 and IgG.
**2**	Normal	Peripancreatic edema and segmental areas of decreased enhancement within the pancreatic body and neck along with a capsule-rim sign. The CBD and pancreatic duct were mildly dilated with narrowing near the pancreatic head.	Stenotic ventral pancreatic duct in the head of the pancreas with irregular and beaded appearance of the main bile duct	Biopsy of the distal bile duct showed fragments of benign biliary mucosa with focal chronic inflammation, detached strips of benign-appearing glandular epithelium with focal mild acute inflammation and was negative for increased IgG-4 plasma cells. Duodenal major papilla biopsy showed duodenal mucosa with chronic acute duodenitis including foveolar metaplasia, increased lamina propria plasma cells, and intraepithelial neutrophils (patch neutrophilic crypitis) and was negative for increased IgG-4 plasma cells.
**3**	Severe extrahepatic biliary dilatation concerning for distal duct obstruction	2.4 cm × 2.4cm mass in the pancreatic head/uncinate process causing extrahepatic biliary dilatation as well as dilation of the gall bladder.	Long segment narrowing of the distal common bile duct consistent with extrinsic compression from known pancreatic head mass.	FNA of peripancreatic lymph node showed polymorphous population of small mature lymphocytes, scattered histiocytes, and occasional lymphoid aggregates. FNA from pancreatic head/uncinate mass are mildly cellular and show scattered cohesive groups of benign ductal cells arranged in honeycomb sheets and lobular proliferation of ducts in the fibromyxoid stroma, which is rich with benign spindle cells, neutrophilic infiltrate, and scattered plasma cells. The neutrophils infiltrate in the ductal epithelium.
**INSPPIRE** ^4^	“Hypoechoic pancreatic parenchyma, diffuse or focal enlargement of the pancreas, a pancreatic mass lesions with or without a dilated CBD in absence of choledocholithiasis.”	“Focal, segmental, or global pancreas enlargement; hypointense pancreas on T1-weighted images; hypointense capsule-like rim on T2-weight images; main pancreatic duct irregularities or stricture; CBD stricture or dilatation of the CBD which tapers toward and enlarged pancreatic head.”		“Acute and chronic inflammatory cell infiltration around pancreas acini or periductular and presence of IgG-4 positive plasma cells with or without pancreas fibrosis.” “More data are needed to determine the utility of major papilla biopsies.”

TUS, transabdominal ultrasound; MRCP, magnetic resonance cholangiopancreatography; ERCP, endoscopic retrograde cholangiopancreatography; EUS, endoscopic ultrasound; CBD, common bile duct; FNA, fine needle aspirate; INSPPIRE, INternational Study Group of Pediatric Pancreatitis: In search for a cuRE.

Endoscopic ultrasound (EUS) was performed due to diagnostic uncertainty, which showed hyperechoic strands, hypoechoic foci, and lobularity throughout the pancreatic parenchyma. Needle core biopsies of the pancreas were consistent with granulocytic epithelial lesions. There was no increase in cells staining with IgG4, but there were scattered plasma cells positive for CD138 and total IgG.

Fecal calprotectin was obtained to screen for inflammatory bowel disease (IBD), and this was normal. He was treated with corticosteroids, leading to improved pain. He completed 2 weeks of corticosteroids, and partial decompression of the biliary duct was confirmed on repeat imaging at 3 months. His subsequent course was complicated by development of insulin-dependent diabetes 1-year post diagnosis.

### Patient 2

A 14-year-old male presented with 2 weeks of abdominal pain, nausea, and vomiting. The patient’s initial evaluation included a lipase of 5627 Units/L, aspartate aminotransferase of 834 Units/L (13–39 Units/L), alanine transaminase of 735 Units/L (7–52 Units/L), and normal TUS. MRCP showed peripancreatic edema consistent with acute pancreatitis. He was initially discharged with improving aminotransferases and lipase but returned 1 week later with worsening abdominal pain. MRCP demonstrated findings of peripancreatic edema and segmental areas of decreased enhancement within the pancreatic body and neck, along with a capsule-rim sign (white arrows in Fig. 1B). The CBD and pancreatic duct were mildly dilated to 8 and 5 mm, respectively, with narrowing near the pancreatic head. In the setting of this repeat admission with elevated transaminases, elevated lipase, and biliary ductal dilation, differential diagnosis of his pancreatitis included gallstone, autoimmune, viral, and idiopathic pancreatitis. Due to concerns for obstruction, endoscopic retrograde cholangiopancreatography (ERCP) was performed, showing a stenotic ventral pancreatic duct in the head of the pancreas with irregular and beaded appearance of the main bile duct. A biliary stent was placed. Biopsy of the bile duct showed chronic inflammation negative for IgG4. Biopsy of the ampulla/major papilla showed chronic active duodenitis, negative for IgG4. Genetic testing revealed a single variant of cystic fibrosis transmembrane conductance regulator (*CFTR*) gene (11TG-5T heterozygous), but no pathogenic variants were detected in chymotrypsinogen gene (*CTRC*), cationic trypsinogen gene (*PRSS1*), or pancreatic secretory trypsin inhibitor gene (*SPINK1*). Autoimmune markers included elevated total IgG and IgG1; low serum IgG4; positive ANA; and smooth muscle antibody with a negative liver-kidney microsome antibody. Liver biopsy showed lobular inflammation and periportal fibrosis with scattered (<10/hpf) IgG4 positive plasma cells, consistent with autoimmune hepatitis (AIH).

Due to mild microcytic anemia, a colonoscopy was performed to screen for IBD, which was normal. The patient was started on pancreatic enzymes following a low fecal elastase of <15 µg/g (normal values >201 µg/g) measurement. Initial treatment consisted of corticosteroids; he was later started on azathioprine as steroids were weaned. The patient’s symptoms resolved with treatment. Follow-up ERCP at 2-months showed decompression of the CBD, and the biliary stent was removed.

### Patient 3

A 4-year-old obese (Z score 4.44) male presented with dark urine, pale stool, and jaundice. Initial labs showed an elevated alanine transaminase of 245 Units/L, aspartate aminotransferase of 299 Units/L, total bilirubin of 5.9 mg/dL (direct 3.9 mg/dL), GGT of 39 Units/L (9–48 Units/L), and alkaline phosphatase of 1141 Units/L. Lipase was within normal limit at 39 Units/L. TUS showed severe extrahepatic biliary dilatation concerning for distal duct obstruction.

MRCP showed a 2.4 cm × 2.4 cm mass in the pancreatic head/uncinate process causing extrahepatic biliary dilatation to 12 mm and dilation of the gall bladder (Fig. 1C). Due to concerns for obstructive cholestasis and pancreatic mass, an ERCP was performed. This revealed a long segment narrowing of the distal CBD consistent with extrinsic compression. A CBD stent was placed. EUS was performed with fine-needle aspiration (FNA) from the pancreatic head/uncinate mass. Biopsy displayed scattered groups of benign ductal cells arranged in honeycomb sheets, and lobular proliferation of ducts in the fibromyxoid stroma rich with benign spindle cells, neutrophilic infiltrate, and scattered plasma cells. The biopsy was negative for IgG4 staining.

Following ERCP, transaminase and bilirubin levels improved and symptoms resolved. Repeat MRCP performed 3 months following stenting demonstrated severe pancreatic atrophy, which had progressed from the prior study, with persistent, but improved narrowing of the CBD. Repeat ERCP at that time showed mild CBD stricture and the patient was restented.

Additional evaluation showed negative autoimmune (antinuclear antibodies, smooth muscle antibody, and liver/kidney microsome antibody) and genetic (Calcium sensor receptor, *CFTR*, *CTRC*, *SPINK1*) work up.

Although the diagnosis was not straightforward, type 2 AIP was diagnosed based on clinical findings of regional disease and histological absence of IgG4-staining of the pancreatic biopsy. The patient was started on corticosteroids due to progression of pancreatic atrophy and concern for progression to pancreatic insufficiency. Follow-up ERCP 3 months following treatment showed resolution of the CBD stricture and the stent was removed. He was noted to have an elevated hemoglobin A1C of 6.2%. It was unclear if this was from endocrine insufficiency or insulin resistance due to obesity.

## DISCUSSION

This case series describes 3 patients with AIP, each with unique features that highlight diagnostic concerns and complications. The patients described showed variable laboratory abnormalities as well as a variety of TUS, MRCP, and ERCP findings. Common themes in presentation included abdominal pain and concern for ductal abnormalities or a pancreatic mass.^1–4^ Comorbid conditions included AIH, diabetes mellitus, and exocrine pancreatic insufficiency (EPI). To our knowledge, coexistent AIH and AIP have not been described in the pediatric literature; however, isolated cases have been described in adults.^5,6^ With AIP, it is important to have a high suspicion for endocrine and exocrine pancreatic insufficiency. In our case series, 1 patient developed insulin-dependent diabetes. Another patient developed EPI as documented by a low fecal elastase. Consideration for coexistent autoimmune conditions is also important; IBD screening occurred in cases in which there was concern, which included colonoscopy or obtaining a fecal calprotectin.^4,7,8^ Fecal calprotectin may be used to rule against IBD in these patients.^8^ Genetic testing for causes of chronic pancreatitis occurred in 2 of the 3 patients described and may be a component of evaluation when diagnosis is uncertain.^4^

The INSPPIRE consortium recently published recommendations regarding a standard diagnostic and therapeutic approach for AIP.^4^ AIP is typically associated with mild and intermittent pain and not always with elevated amylase and lipase.^4^ IgG4 may be elevated in only 20% of children with AIP, and its use as a criterion is questioned.^1,2,4^ A TUS typically serves as initial imaging in pancreatitis and obstructive jaundice; however, suspicion for AIP should prompt MRCP.^4,9^ MRCP and histologic findings are outlined in Table 1, and the presence of these findings should raise suspicion for AIP.^2,4,10^ Tissue diagnosis should ideally be obtained before initiating therapy for AIP per INSPPIRE guidelines; however, barriers exist due to limited number of skilled pediatric endoscopists in EUS or ERCP.^4,9^

MRCP, EUS, endoscopic biopsy/FNA, and ERCP are available at our center. CBD stricture was present on MRCP in each case described, as well as additional characteristic imaging findings (Table 1). A needle core biopsy in patient 1 showed features consistent with AIP.^2,7^ The duodenal and distal bile duct biopsies in patient 2 showed features of an autoinflammatory condition and aided in the diagnosis for this patient. Biopsies of the papilla have not been evaluated in children and may be a consideration for further evaluation.^7^ All samples were negative for increased staining of IgG4, which is consistent with previous reports in the literature in children.^2,7^ While distinguishing the subtypes of AIP in children can be challenging and is based on adult criteria, the negative staining for IgG4 suggests type 2 AIP in patients 1 and 3.^2,4^

Corticosteroids are first-line treatment for AIP serving to both improve symptoms and potentially prevent complications including biliary or pancreatic duct strictures.^2,4,10^ In our patients, presenting symptoms resolved in all with corticosteroid administration. Follow-up imaging showed resolution of CBD dilatation in all patients. Relapse can occur and may require repeat dosing of corticosteroids and the addition of an immunomodulator.^2,4,10^

In conclusion, this review demonstrates differences in presentation and diagnostic considerations in pediatric AIP. Further, this article highlights the importance of screening for comorbid conditions such as EPI and Type 3c DM. AIH was seen in one patient along with AIP. AIP should be a diagnostic consideration in patients with characteristic MRCP findings or concern for ductal changes or inflammatory mass on imaging.
